# Treatment ineffectiveness towards *Haemonchus contortus* is highly prevalent in sheep and goat farms of North-Eastern Italy

**DOI:** 10.1186/s12917-024-04347-7

**Published:** 2024-10-30

**Authors:** Anna Maurizio, Giorgia Dotto, Antonia Fasoli, Francesco Gaio, Sara Petratti, Alice Pertile, Cinzia Tessarin, Erica Marchiori, Debora Dellamaria, Jaroslav Vadlejch, Rudi Cassini

**Affiliations:** 1 Department of Animal Medicine, Production and Health, University of Padova, Viale dell’Università, 16, Legnaro, PD 35020 Italy; 2 Veterinary practicioner, Trento, Italy; 3 Veterinary practicioner, Verona, Italy; 4 Veterinary practicioner, Vicenza, Italy; 5 Istituzione Zooprofilattico Sperimentale delle Venezie, Viale dell’Università, 10, Legnaro, PD 35020 Italy; 6 Department of Zoology and Fisheries, Faculty of Agrobiology, Food and Natural Resources, Czech University of Life Sciences Prague, Kamycka 129, Prague Suchdol, 165 00 Czech Republic

**Keywords:** Small ruminants, Gastrointestinal nematodes, Control, Faecal egg count reduction test, Anthelmintic resistance

## Abstract

**Background:**

Anthelmintic resistance (AR) is a global threat to grazing livestock farming. In Italy, anthelmintic efficacy remains high compared to other European countries, but many parts of the country haven’t been investigated yet. Local veterinary practitioners from Trentino and Veneto regions reported suspected inefficacy towards anthelmintic drugs in some of their farms, prompting a study on AR in sheep and goat farms of northern Italy. The study aimed to assess anthelmintic effectiveness using genus-specific faecal egg count reduction tests (FECRT), to detect differences in treatment response among nematode genera involved in the infection.

**Results:**

Twelve farms (6 sheep and 6 goat farms) were included based on clinical suspicion of AR. Treatments were carried out with either benzimidazoles (BZ) or macrocyclic lactones (ML) Treatment was effective in 3/6 goat trials, with reduced effectiveness to BZ in two farms and to ML the last one. In sheep farms (6/6), effectiveness was consistently and more severely insufficient. Ineffectiveness was particularly high towards *Haemonchus contortus*, while *Oesophagostomum*/*Chabertia* maintained susceptibility in nearly all trials. *Trichostrongylus*/*Teladorsagia* exhibited intermediate results.

**Conclusions:**

This study reveals diminished efficacy of both BZ and ML in small ruminant farms in north-eastern Italy, an area previously lacking data on the topic, except for goats in South Tyrol. Variability in treatment responses among nematode genera support suspicions of AR, and further concerns are raised by the prevalence of treatment ineffectiveness against the highly pathogenic *Haemonchus contortus*. This finding underscores the urgent need for comprehensive AR monitoring in the area and improved management practices to prevent further resistance development and protect livestock health.

## Introduction

Anthelmintic resistance (AR) is one of the major threats that grazing livestock farming is currently facing worldwide. The use of anthelmintic drugs as sole control measure against gastrointestinal nematodes (GIN) is an established practice [1], well described also in Italy [2–5]. Resistance development is a natural and inevitable response of organisms when exposed to a selective pressure [6], in this case to anthelmintics. However, despite the numerous reports of AR throughout Europe in the past three to four decades, Italy seemed to be less affected by the spread of the phenomenon compared to other countries, all-the-more considering the country’s significant research efforts for AR detection [7]. In 2020 members of the EU COST Action COMBAR (Combatting Anthelmintic Resistance in Ruminants; https://www.combar-ca.eu) from 26 European countries created an online database (publicly available at https://osf.io/7jzuf, last updated on 28/06/2021) including published and unpublished AR research in domestic ruminants throughout the continent. In this database data from 20 published and unpublished studies are available for Italy, but only one of them [8] is focused on northern Italy (on goats of the South Tyrol province), while only two more include ≤ 5 farms each from northern Italy [9, 10] (on sheep and cattle respectively). One additional existing research on AR in goats from north-western Italy [3] is absent in the database. Research efforts are therefore concentrated in the south of the country, where most of the Italian small ruminant production (especially dairy sheep) is concentrated [11]. Northern Italy is dominated by intensive poultry, pig and cattle farming, but small ruminants are also reared within peculiar farming systems. To preserve these production and farming systems, mainly based on grazing and therefore at risk for GIN infection, attention should be given to the problem posed by AR in GIN control. Scientific literature suggest that AR is already sporadically present in Italy, although anthelmintic efficacy is still quite high. To preserve the current condition, close monitoring should be implemented in order to ensure prompt action in case of onset of resistance [12]. Suspects of anthelmintic inefficacy were informally raised by local veterinarians from north-eastern Italy, more specifically Trentino province and Veneto region, and led to this study, which aimed at assessing the effectiveness of anthelmintics in sheep and goat farms in a part of northern Italy up-to-now never investigated. Differences in efficacy among GIN genera were also assessed using a genus-specific FECRT approach based on identification of cultured larvae [13].

## Materials and methods

### Study area

The study investigates the north-eastern part of Italy, more specifically Veneto region and the autonomous province of Trentino. The area includes the flat and industrialized Po valley, where the climate is humid-temperate, facing the Adriatic Sea on the south and surrounded on the north by the Alps, where the climate is more cold and wet during the summer. In that area, sheep farming is mainly associated with meat production, and it is typically transhumant, with movement of sheep from the lowlands during the winter to the mountains in the summer. In the alpine area, smaller sheep and goat farms serve both environmental maintenance and local production purposes, and are often part-time managed and with limited economic resources [5, 14]. Grazing is alternated between pasture areas at lower altitudes in spring and autumn and summer pastures at higher altitudes in summer. A barn is usually available for stalling during the winter. Conversely, within the Po valley, dairy goats are primarily kept in more modern and intensive farms, often associated with on site cheese production [15]. These goats are provided with either continuous access to grazing areas or restricted access for a set number of hours per day. However, in the area, some farms also rear goats exclusively indoors.

### Sample collection and anthelmintic treatment

The study was carried out between October 2021 and July 2022 in sheep and goat farms of north-eastern Italy. Local veterinarians were contacted and they selected the farms to include based on suspects of anthelmintic inefficacy, availability of farmers, risk for GIN infection (access to pasture was a prerequisite) and a history of at least one anthelmintic treatment per year. The suspect of treatment failure was based on clinical signs (primarily rough coat and low body condition score) as well as the presence of parasites (mainly *Haemonchus contortus*) during necropsies. FECRT trials were conducted in concomitance with the anthelmintic treatment planned by each farm veterinarian, no less than 6 months after the previous anthelmintic treatment. Faecal samples were collected from ten adult females right before treatment administration (D0) and then again from the same animals 11–17 days later (D14). The number of days intercurring between the two samplings was influenced by the drug used, according to the guidelines available at the time of the study [16–18]. For each trial the anthelmintic drug was independently selected by the local veterinarian (the list of anthelmintics used is available in the Results section) and administered at the dosage recommended by the package leaflet in sheep, while double the dosage of sheep was administered in goats, as recommended in the scientific literature [19]. Dosage was based on visual estimation of animals’ body weight, but few random animals were weighted with a dynamometer and a weighing harness to confirm the accuracy of the estimation.

Faecal samples were maintained under cold chain until laboratory analyses, which were carried out within 48 h from collection at the Parasitology Laboratory of the Department of Animal Medicine, Production and Health of the University of Padova. For genus-specific FECRT pooled samples were employed, as previously tested in Maurizio et al. [13]. Two pooled samples at both D0 and D14 were composed for each trial, using 6 g of faeces from five individuals each and maintaining the same division of animals in sub-groups at D14. Pooled samples were thoroughly homogenized and used to perform coproculture.

### Laboratory analysis

Faecal Egg Counts were carried out on individual samples. For each sample, 5 g of faeces were analyzed using the McMaster technique [20] with a limit of detection of 20 EPG, as described in Maurizio et al. (2024). After filling the McMaster chambers and waiting about 5 min, parasitic eggs were counted in both chambers, although for the purposes of this study only strongylid-type (which we refer to as GIN) eggs were considered.

The faeces of each pool (30 g) were cultured at 26° C for 7 days to obtain third-stage larvae (L3s) for morphological identification of GIN genera. Vermiculite was added to faeces to facilitate ventilation and prevent fungal contamination, and faeces were kept moist throughout the incubation period by adding water daily. L3s were then collected using the Baermann technique and stored at + 4^o^ C until identification. The first 50 larvae recovered for each pool were identified as *Haemonchus* (type T1), *Trichostrongylus* / *Teladorsagia* (T2), *Oesophagostomum* / *Chabertia* (T3) and *Bunostomum* (T4) by keys currently in use at the Laboratory of Parasitology of the University of Padova. These keys are based on data proposed by several authors [20–22] and experiences gained at our lab, as described in Maurizio et al. [13]. If fewer than 50 larvae were present, all were identified. The NIS-Elements imaging software (Nikon Corporation, Japan) was used for morphometric measurements to support larval identification.

### Data analysis

For each trial, FECR and 90% confidence intervals (90% CI) for both general and genus-specific FECRT were obtained. Calculation [23] and interpretation [24] followed the latest WAAVP guidelines [25]. The minimum efficacy target was set at 90%, while the expected efficacy was 95%, in line with the previous guidelines [16]. The classification was as follows:

- Resistant (R) when the upper limit of the 90% CI (CI_U_) < 95%.
- Low resistant (LR, a sub-category of the previous) when the lower limit of the 90% CI (CI_L_) ≥ 90%;
- Inconclusive (INC) when CI_U_ ≥ 95% and CI_L_ < 90%;
- Susceptible (S) when CI_U_ ≥ 95% and CI_L_ ≥ 90%.

For genus-specific FECRT, the relative proportions of each genus, identified through larval examination (100 larvae identified on D0 and 100 on D14, using two pooled samples for each trial at both time points), were converted into absolute egg counts, following the method outlined by Maurizio et al. [13].

## Results

Anthelminthic efficacy was tested by FECRT in 6 goat and 6 sheep farms from Veneto (Verona, Vicenza and Belluno Provinces) and Trentino (Trento Province) regions of north-eastern Italy. The main features of each farm are presented in Table 1. In half of the goat farms anthelmintic treatment is administered once a year (G1_BZ, G3_BZ and G5_BZ), and in the remaining twice a year, while all sheep farms treat all animals twice a year.

**Table 1:** Main features of the farms involved in the trials. Size refers to the number of animals > 6 months old, treatment/year to the number of anthelmintic treatments administered per year

Species	ID	Province	Size	Breed	Farm type
Goat	G1_BZ	Verona	35	Chamois Coloured goat	Small pre-alpine farm, daily grazing in surrounding pastures
	G2_BZ	Trento	22	Passiria	Mountain farm rearing a local breed, grazing in surrounding pastures
	G3_BZ	Vicenza	45	Chamois Coloured goat	Semi-intensive pre-alpine farm with adjacent confined pasture
	G4_ML	Trento	27	Passiria	Mountain farm rearing a local breed, alpine grazing during the summer
	G5_BZ	Vicenza	200	Saanen	Semi-intensive farm of the Po valley with adjacent confined pasture
	G6_ML	Trento	41	Passiria	Same as G2_ML
Sheep	S1_ML	Belluno	20	Lamon	Mountain farm rearing an endangered local breed, grazing in surrounding pastures
	S2_ML	Belluno	23	Lamon	Same as S1_ML
	S3_ML	Trento	80	Biellese-Bergamasca crossbreeds	Transhumant within the mountain valley
	S4_ML	Trento	250	Several (cross)breeds	Transhumant from the Po valley (winter) to the mountains (summer)
	S5_BZ	Verona	3000	Bergamasca	Same as S3_ML
	S6_BZ	Trento	15	Biellese and Biellese crossbreeds	Mountain farm, grazing in surrounding pastures

For FECRT, the used drugs were benzimidazoles and avermectines, as described in Table 2. Treatment was effective in 3/6 goat trials, while a low resistance to oxfendazole emerged in one farm (G5_BZ). The two remaining trials (G2_BZ and G4_ML), with a FECR of 78.1% and 90.8%, highlighted a reduced effectiveness towards albendazole and ivermectin respectively. In sheep farms, effectiveness was reduced in all trials, with a FECR ranging from 91.1% to − 62.2% (the negative value indicating an increase in FEC at D14 compared to the D0 counts).

**Table 2:** FECR and 90% confidence intervals in trials of the study. FEC at D0 and D14 refer to total counted eggs among the 10 animals

	ID	Drug	Class	Admin. route	FEC		FECR (%)	90% CI	Efficacy
					D0	D14			
Goat	G1_BZ	Fenbendazole^a^	BZ	PO	613	7	98.9	*97.9–99.4*	S
	G2_BZ	Albendazole^b^	BZ	PO	260	57	78.1	*73.7–82.1*	R
	G3_BZ	Albendazole^c^	BZ	PO	1600	0	100	*99.8–100*	S
	G4_ML	Ivermectin^d^*	ML	SC	173	16	90.8	*86.4–93.7*	R
	G5_BZ	Oxfendazole^e^	BZ	PO	2386	194	91.9	*90.9–92.7*	LR
	G6_ML	Ivermectin^d^*	ML	SC	262	0	100	*98.9–100*	S
Sheep	S1_ML	Ivermectin^f^	ML	SC	937	795	15.2	*13.3–17.2*	R
	S2_ML	Ivermectin^f^	ML	SC	428	694	-62.2	*--*	R
	S3_ML	Ivermectin^f^	ML	SC	950	181	81.0	*78.7–82.9*	R
	S4_ML	Eprinomectin^g^	ML	pour-on	660	170	74.4	*71.3–76.9*	R
	S5_BZ	Albendazole^b^	BZ	PO	1361	121	91.1	*89.7–92.3*	R
	S6_BZ	Albendazole^b^	BZ	PO	283	62	78.1	*73.7–81.8*	R

^a^ Panacur 2.5%^®^, MSD; ^b^ Sverminator^®^, Fatro; ^c^ Contruerme^®^, Izo; ^d^ Ivomec plus^®^, Boerhinger Ingelheim; ^e^ Oxfenil^®^, Virbac; ^f^ Ivomec Ovini^®^, Boerhinger Ingelheim; ^g^ Eprinex Multi^®^, Boerhinger Ingelheim

* Ivermectin + Clorsulon. BZ = benzimidazoles; ML = macrocyclic lactones; Admin. route = administration route; SC = subcutaneous injection; PO = peroral. --, not calculable. S = susceptible; LR = low resistant; R = resistant

For each trial, FECRT was calculated both overall and specifically for each previously described type. Results of the genus-specific FECRT (Table 3) indicated clear differences in terms of reduced effectiveness between the genera considered. T1 (*Haemonchus*) displayed the worst results in both sheep and goats, obtaining the lowest reduction in most trials or even increasing in three trials. T2 (*Trichostrongylus*/*Teladorsagia*) had slightly better results, but treatment effectiveness was still insufficient in five trials and in one case (S4_ML) it was even lower than *Haemonchus*. Susceptibility was instead maintained by M3 (*Oesophagostomum*/*Chabertia*) in 11/12 trials.

**Table 3:** FECR and 90% confidence intervals in trials of the study, calculated for overall strongylids and for each type (T1 = *Haemonchus*; T2 = *Trichostrongylus*/*Teladorsagia*; T3 = *Oesophagostomum*/*Chabertia*)

	ID	Genus	FEC		FECR%	90% CI	Efficacy
			D0	D14			
Goat	G1_BZ	**Overall strongylids**	**613**	**7**	**98.9**	***97.9–99.4***	**S**
		*T1*	116.5	2.6	97.8	*94.0–99.0*	S
		*T2*	312.6	0.5	99.8	*98.7–99.9*	S
		*T3*	183.9	3.9	97.9	*95.2–99.0*	S
	G2_BZ	**Overall strongylids**	**259.5**	**56.5**	**78.2**	***73.7–82.1***	**R**
		*T1*	31.1	50.1	-61.0	*--*	R
		*T2*	23.4	6.4	72.7	*55.4–84.5*	R
		*T3*	205.0	0.0	100	*98.6–100*	S
	G3_BZ	**Overall strongylids**	**1600**	**0**	**100**	***99.8–100***	**S**
		*T1*	1376.0	0.0	100	*99.8–100*	S
		*T2*	0.0	0.0	--	*--*	--
		*T3*	224.0	0.0	100	*98.7–100*	S
	G4_ML	**Overall strongylids**	**173**	**16**	**90.8**	***86.4–93.7***	**R**
		*T1*	100.3	12.6	87.5	*80.9–91.8*	R
		*T2*	43.3	3.4	92.1	*82.2–96.3*	INC
		*T3*	29.4	0.0	100	*90.6–99.8*	S
	G5_BZ	**Overall strongylids**	**2386**	**194**	**91.9**	***90.9–92.7***	**LR**
		*T1*	811.2	181.2	77.7	*75.1–80.0*	R
		*T2*	1336.2	12.8	99.0	*98.5–99.4*	S
		*T3*	238.6	0.0	100	*98.8–100*	S
	G6_ML	**Overall strongylids**	**262**	**0**	**100**	***98.9–100***	**S**
		*T1*	209.6	0	100	*98.6–100*	S
		*T2*	49.1	0	100	*94.2–99.9*	S
		*T3*	87.3	0	100	*96.7–99.9*	S
Sheep	S1_ML	**Overall strongylids**	**937**	**795**	**15.2**	***13.3–17.2***	**R**
		*T1*	815.2	755.3	7.4	*6.0–9.0*	R
		*T2*	75.0	31.8	57.6	*48.1–66.5*	R
		*T3*	469	8.0	83.0	*72.0–89.9*	R
	S2_ML	**Overall strongylids**	**428**	**694**	**-62.1**	***--***	**R**
		*T1*	340.7	651.1	-91.1	*--*	R
		*T2*	69.9	42.9	38.6	*29.7–48.5*	R
		*T3*	17.5	0.0	100	*85.0–99.7*	INC
	S3_ML	**Overall strongylids**	**949.5**	**181**	**80.9**	***78.7–82.9***	**R**
		*T1*	862.3	181.0	79.0	*76.6–81.2*	R
		*T2*	9.7	0.0	100	*75.6–99.5*	INC
		*T3*	77.5	0.0	100	*96.3–99.9*	S
	S4_ML	**Overall strongylids**	**660**	**170**	**74.2**	***71.3–76.9***	**R**
		*T1*	587.4	144.0	75.5	*72.4–78.3*	R
		*T2*	19.8	22.6	-13.9	*--*	R
		*T3*	52.8	3.5	93.4	*85.1–96.9*	INC
	S5_BZ	**Overall strongylids**	**1361**	**121**	**91.1**	***89.7–92.3***	**R**
		*T1*	1234.7	119.8	90.3	*88.8–91.6*	R
		*T2*	70.2	1.2	98.3	*93.1–99.4*	S
		*T3*	56.1	0.0	100	*94.9–99.9*	S
	S6_BZ	**Overall strongylids**	**282**	**62**	**78.0**	***73.7–81.8***	**R**
		*T1*	49.8	58.6	-17.8	*--*	R
		*T2*	176.9	3.4	98.1	*95.4–99.1*	S
		*T3*	55.3	0.0	100	*94.8–99.9*	S

FEC at D0 and D14 refer to total counted eggs. --, not calculable. S = susceptible; INC = inconclusive; LR = low resistant; R = resistant

## Discussion

This study originated from a suspect of failed treatments raised by local veterinary practitioners in north-eastern Italy. Extremely limited epidemiological data are available in the area in the past decade, confined to the South Tyrol province in sheep and goats [5] or to goats in Veneto and Friuli – Venezia Giulia regions [4]. Only one study was carried out to assess anthelmintic efficacy in small-scale goat farms in the mountainous province of South Tyrol [8]. The purpose of the present study was to verify the effectiveness of anthelmintics in sheep and goat farms of Trentino province and Veneto region, aiming at detecting evidence of anthelmintic resistance in the study area rather than at estimating the true prevalence of AR. Hence, local veterinarians selected farms with a history of clinically suspected treatment failure.

FECRT results confirmed anthelmintic inefficacy in both goats (2/6 farms, plus 1/6 with only mild inefficacy) and sheep (6/6 farms) farms, to both benzimidazoles and macrocyclic lactones in each species. The term “inefficacy” is here preferred to “resistance” for the broad range of confounding factors (pharmacokinetics, pharmacodynamics, host and parasite factors, as well as technical factors) which might influence the results [26]. For instance, when pour on formulations are used, underdosing could be due to animals licking off the treatment, which might not be fully controlled in a field setting. It is although inevitable that ineffective treatments represent a strong selective pressure for resistance, which, if not already present, is undeniably a huge risk for these farms. AR is already reported by FECRT-based studies in goats from neighboring regions: in Lombardy region AR was reported in about 30% (4/15) of the investigated flocks against both benzimidazoles and macrocyclic lactones and associated to resistance of *Trichostrongylus*/*Teladorsagia* (for BZ and ML) and of *Haemonchus* (for ML). In South Tyrol FECRT were carried out both during routine treatments (8 trials) and under controlled conditions (15 trials) [8]. Insufficient efficacy was more common during routine treatments (4/8) than under controlled conditions (2/15), but in both conditions it was detected against both macrocyclic lactones and benzimidazoles and it was especially associated to *Haemonchus* and *Trichostrongylus* genera. No other studies on sheep are available in northern Italy, despite a population of almost half a million sheep (National Data Bank - NDB on 30th June 2023) [27]. Only Geurden et al. [9] conducted FECRT on 10 farms from six Italian provinces, one of which based in the North, but data from north and south were not presented separately so no extrapolation is possible.

We did not carry out an in-depth questionnaire on nematode control practices, but informal conversations revealed a management comparable to what previously reported in the area for goats [4]: anthelmintic treatments carried out on the whole group once to twice a year with visual estimation of the weight, almost no FEC monitoring, no quarantine of newly introduced animals, and drug rotation present almost only when more than a treatment per year is performed. In Maurizio et al. [4] goats were treated during the dry period (November to January) and if necessary again in spring/summer. This same strategy was employed by many goat farms in this study, while farms in the alpine area (both sheep and goat farms) tended to treat twice a year, at the beginning (around April) and at the end (around October) of the grazing season, similarly to what reported by Lambertz et al. [5] in a neighboring province (South Tyrol).

Despite the selection of farms based on suspicion of anthelmintic treatment failures, the high level of inefficacy obtained in this study was unexpected, but the subsequent genus-specific FECRTs supports the results. The genus-specific approach highlighted a non-homogeneous response to treatment among genera, suggesting that some genera might be indeed developing resistance. *Haemonchus contortus* (T1) seemed to be mainly involved in the phenomenon and its dominance in the GIN community at the D0 samplings (T1 was the more abundant genus in 5/6 sheep farms) suggest that this process might be ongoing since years. In the broader European context, *Haemonchus* together with *Trichostrongylus* and *Teladorsagia* are the most frequently genera reported in studies where resistance to BZ, ML, levamisole, and multi-drug AR has been detected [7]. The limitations of the genus-specific FECRT approach have been discussed in a previous article [13], but it should be highlighted that also in the present study it was not possible to reach a count of 200 eggs for each type in each trial, thus increasing the uncertainty of the results. However, limiting the observation to *Haemonchus contortus* (T1), in 8/12 trials this minimum requirement was fulfilled.

Farms included represent all the production systems of the area (see subsection 2.1), but FECRT results don’t appear to be associated with any system in particular, although the sample size per type is certainly too low. One element of interest is the potential for exchange of resistant nematodes between farms. Except for G3_BZ and G5_BZ, all the other farms use shared pastures, at the same time or subsequently. Animals from several farms might be grazed together during the summer in alpine pastures, and the routes of transhumant sheep are often forced by orography or buildings, crops and villages, so they overlap in some parts. Transmission of resistant GIN strains among farms in this context certainly represent a risk, although for farms with high levels of resistance (e.g., S1_ML and S2_ML) shared grazing could be a beneficial source of susceptible parasites. These dynamics are extremely complex and require specific investigations to be unraveled. Although they might have a significant impact on the spread of AR in the area, they also somehow complicate the communication and education around AR management and prevention, whose uptake of basic principles is still limited.

## Conclusions

The present study provides strong indication of reduced efficacy of both benzimidazoles and macrocyclic lactones (moxidectin not tested) in small ruminant farms of north-eastern Italy, mainly associated to the genus *Haemonchus*. The study was carried out in farms where treatment inefficacy was suspected on a clinical basis, but the process might be subtly ongoing also in other farms. The diagnosis of AR cannot be confirmed by a single FECRT trial but widespread ineffective treatments certainly represent a strong selective pressure towards resistant GIN. A wider non-biased survey could be useful to understand the actual extent of the phenomenon and the factors promoting its spread in the area.

## Data Availability

The datasets generated and/or analysed during the current study are available from the corresponding author on request.
